# The importance of monitoring a new antibiotic: ceftazidime/avibactam usage and resistance experience from England, 2016 to 2020

**DOI:** 10.2807/1560-7917.ES.2025.30.14.2400399

**Published:** 2025-04-10

**Authors:** Rebecca L Guy, Katie L Hopkins, Emma L Budd, Kate Wilson, Holly Fountain, Danièle Meunier, Rachel Pike, Sarah M Gerver, Roshina Gnanadurai, Colin S Brown, Susan Hopkins, Berit Muller-Pebody

**Affiliations:** 1 AMR & HCAI Division, UK Health Security Agency, London, United Kingdom; 2 Antimicrobial Resistance and Healthcare Associated Infections (AMRHAI) Reference Unit, UK Health Security Agency, London, United Kingdom; 3 Bacterial Reference Department, UK Health Security Agency, London, United Kingdom

**Keywords:** AMR, carbapenemase, new antimicrobial, emerging resistance, AMS, AMU, ceftazidime/avibactam, Enterobacterales, CPE

## Abstract

**Background:**

Ceftazidime/avibactam, launched in the United Kingdom (UK) in March 2017, is an antibiotic against multidrug-resistant Gram-negative pathogens. It was selected for the government’s subscription model pilot, for incentivising new antibiotic development, which began in December 2020.

**Aim:**

Ahead of the pilot, we assessed ceftazidime/avibactam testing, resistance (2016−2020) and usage (2017−2020) levels in England, as baselines for future surveillance.

**Methods:**

From routine surveillance samples, we retrieved reported ceftazidime/avibactam resistance categorisation. From reference laboratory samples, we reviewed minimum inhibitory concentration (MICs) and molecular data. Among surveillance samples, per cent resistance was estimated. Referred samples’ MICs, by carbapenemase gene presence, were investigated. Ceftazidime/avibactam hospital use was measured in defined daily doses (DDDs).

**Results:**

Overall, 6.3% (4,200/66,914; 95% confidence interval (95%CI): 6.1–6.4%) of surveillance-reported ceftazidime/avibactam-tested Gram-negative bacteria were resistant. Percentage resistance per bacterial species varied over time, somewhat stabilising as testing was established, with between April 2019 and March 2020, 1.3% *Escherichia coli* (288/22,736; 95%CI: 1.1−1.4%), 12.6% *Pseudomonas aeruginosa* (690/5,495; 95%CI: 11.7−13.5%) and 6.1% of *Klebsiella pneumoniae* (314/5,179; 95%CI: 5.4−6.7%) being resistant. For 8,437 referred Enterobacterales, MIC determination found 11.5% (968/8,437; 95%CI: 10.8–12.2%) resistant. Among resistant isolates, 89.3% (864/968; 95%CI: 87.1–91.1%) had metallo-β-lactamase (MBL) genes. Of 908 MBL-negative isolates, producing ≥ 1 non-metallo-carbapenemase(s), 2.1% (19/908; 95%CI: 1.3–3.2%) were resistant. Since March 2017, 69.5% (105/151) of English National Health Service Trusts used ceftazidime/avibactam. Monthly usage progressed from 21 to 744 DDDs in March 2020.

**Conclusion:**

For appropriate treatment, carbapenemase gene detection and variant identification in ceftazidime/avibactam surveillance matters. Detecting emerging resistant pathogens and preventing spread within healthcare settings requires vigilance.

**Key public health message**

**What did you want to address in this study and why?**

In England a pre-payment subscription model pilot incentivising antibiotic development began in December 2020, with ceftazidime/avibactam selected as one of the pilot antibiotics. For future assessment of this model, we wanted to estimate levels of ceftazidime/avibactam use, susceptibility testing and resistance prior to the pilot start. As resistance can be conferred by certain carbapenemases, carbapenemase genes were studied in some resistant bacteria.

**What have we learnt from this study?**

We learned that levels of ceftazidime/avibactam use and susceptibility testing seem to have increased in the lead up to the pilot. Moreover, low levels of resistance were observed in the country, coinciding in some cases, with the presence of certain carbapenemase genes in bacteria. We also found that suspicion of resistance development in a patient during treatment with ceftazidime/avibactam has been noted.

**What are the implications of your findings for public health?**

Our analysis highlights the importance of carbapenemase gene identification before ceftazidime/avibactam use. There is also a potential risk of developing resistance while on treatment. Best practice is to establish individual patient treatment plans using microbiology and surveillance data; it is also important for microbiologists and clinicians to identify and report emerging resistance to ceftazidime/avibactam in bacteria to prevent its spread.

## Introduction

Ceftazidime/avibactam is a β-lactam/β-lactamase inhibitor combination, which was licensed by the European Medicines Agency (EMA) in June 2016 and subsequently launched in the United Kingdom (UK) in March 2017. Ceftazidime/avibactam is licensed for the treatment of complicated intra-abdominal infections, complicated urinary tract infections and hospital-acquired pneumonia caused by Gram-negative bacteria in adults (aged ≥ 18 years) and paediatric patients aged 3 months and older in the UK. It is also licensed for use against infections caused by multidrug-resistant Gram-negative bacteria with limited treatment options, such as those caused by extended-spectrum β-lactamase (ESBL)-producing or carbapenem-resistant Enterobacterales (CRE) and carbapenem-resistant *Pseudomonas aeruginosa* [1]. Ceftazidime/avibactam is active against class A, C and some class D β-lactamases including *Klebsiella pneumoniae* carbapenemase (KPC) and oxacillinase (OXA)-48-like carbapenemases but not against the metallo-β-lactamases (MBL), including New Delhi metallo-β-lactamase (NDM), Verona integron-mediated metallo-β-lactamase (VIM) and imipenemase (IMP) [2].

In June 2018, the European Centre for Disease Prevention and Control published a risk assessment highlighting the emergence of resistance to ceftazidime/avibactam, which concluded ceftazidime/avibactam-resistant CRE pose a public health threat due to the likelihood of further spread within healthcare settings with potential adverse outcomes for patients [3]. Investigations into the underlying resistance mechanisms have identified several causes including amino acid substitutions within critical sites in KPC and other β-lactamases, increased *bla*_KPC_ gene copy number, point mutations within penicillin-binding proteins (PBPs), increased efflux and porin defects leading to impermeability [4-6].

Global data from the International Network for Optimal Resistance Monitoring (INFORM) surveillance programme (2015–2017) indicated that ceftazidime/avibactam had excellent activity against meropenem-non-susceptible Enterobacterales that were identified as carbapenemase positive/MBL negative (99.8%) and carbapenemase negative/MBL negative (95.9%) with susceptibility rates higher than for comparator agents colistin and tigecycline [6]. The UK Health Security Agency’s (UKHSA, formerly Public Health England), Antimicrobial Resistance and Healthcare Associated Infections (AMRHAI) Reference Unit in Colindale, London, began screening all Enterobacterales referred for minimum inhibitory concentration (MIC) determination (primarily due to carbapenem resistance) against ceftazidime/avibactam in July 2015. Over the subsequent 12 months, this initiative revealed susceptibility rates exceeding 95% for Enterobacterales producing class A or D carbapenemases and ESBL or AmpC producers [7]. Hospital outbreaks in the United States and Europe, caused by extensively drug-resistant *K. pneumoniae* including resistance to ceftazidime/avibactam [8,9] as well as emergence of resistance during treatment with ceftazidime/avibactam [10] have been described in 2020 and 2022 respectively. This raises the concern that the increase in usage of ceftazidime/avibactam may subsequently lead to an increase in resistance to this new antibiotic.

Additionally, ceftazidime/avibactam was selected for the pilot of an innovative national approach to preserving antibiotics for the future by incentivising production of new antimicrobials as part of a subscription model for antimicrobials [11] where the National Health Service (NHS) England paid pharmaceutical companies a fixed annual fee for two selected antimicrobials (ceftazidime/avibactam and cefiderocol), based on a health technology assessment of their value rather than the volumes of antimicrobial used [11]. The scheme was launched in December 2020 [12], and following the successful pilot, the first commercial subscription style contracts de-linked from use were awarded in July 2022.

To inform activities aimed at conserving the effectiveness of ceftazidime/avibactam and to provide a baseline for both monitoring and further evaluating the UK’s subscription model, this study, which was conducted in England ahead of the model pilot implementation, had two objectives. The first was to determine changes in levels of ceftazidime/avibactam susceptibility testing and resistance between 1 April 2016 and 31 March 2020. The second was to evaluate the evolution of prescribing of this last-line therapeutic agent from its initial launch in the country, in March 2017 up to 31 March 2020.

## Methods

### Susceptibility testing data sources

#### Routine national diagnostic laboratory surveillance

All laboratory records including ceftazidime/avibactam susceptibility data were extracted from UKHSA’s Second Generation Surveillance System (SGSS) from 1 April 2016 to 31 March 2020. This system captures national routine laboratory surveillance data on infectious diseases and antimicrobial resistance from 98% of diagnostic laboratories in England (both NHS and private laboratories). Reporting to SGSS includes all notifiable organisms, clinically relevant specimens as well as results for any specimen where a susceptibility test has been performed [13]. In a survey conducted in 2018 (response rate 94%; 113/120), 98% of participating diagnostic laboratories indicated that they report antibiotic susceptibility data to SGSS, and 95% of responding laboratories reported that they used British Society for Antimicrobial Chemotherapy (BSAC) or European Committee on Antimicrobial Susceptibility Testing (EUCAST) breakpoints. Since March 2016, the SGSS set-up has been adapted, with ceftazidime/avibactam figuring among the antibiotics to choose from, to report susceptibility testing determined by local laboratories and results thereof (Susceptible ‘S’ or Resistant ‘R’).

Infection episodes were deduplicated where patients had more than one positive sample taken that yielded growth of the same pathogen. However, if such samples on the same date had differing susceptibility results, the resistant antimicrobial result was selected over the susceptible one to define the resistance profile of that episode.

#### National reference microbiology laboratory data

Referral to UKHSA’s AMRHAI Reference Unit is voluntary with NHS and private diagnostic laboratories encouraged to submit bacteria isolated from hospital in- and out-patients, and general practice patients of all ages who meet referral criteria outlining exceptional resistance [14]. These bacteria are submitted for molecular detection of antimicrobial resistance mechanisms (primarily acquired carbapenemase genes) and/or phenotypic antimicrobial susceptibility testing. Changes to the AMRHAI referral criteria that were applied during the timeframe of this study are: (i) from October 2018, antimicrobial susceptibility testing was no longer performed on bacterial isolates representing gut colonisation unless specifically requested by a clinician; (ii) from January 2019, laboratories were no longer encouraged to submit all carbapenemase-producing organisms, only those from sterile sites unless confirmation of carbapenemase detection result and/or antimicrobial susceptibility testing was required and (iii) from April 2019, a charge was implemented for NHS laboratories for carbapenemase gene detection if not previously screened for KPC, OXA-48-like, NDM or VIM genes, or diagnostic laboratory results were not provided on the referral form. This coincided with the increase in capacity and capability for local diagnostic laboratory carbapenemase detection, captured by the routine surveillance in SGSS.

Ceftazidime/avibactam MIC data of Enterobacterales isolates from patient samples submitted by English diagnostic laboratories to UKHSA’s AMRHAI Reference Unit were extracted for 1 April 2016–31 March 2020. Demographic data of patients were not available at the time of the study. Duplicate isolates from the same patient that shared the same external reference number, sample site and date of isolation were excluded from the analysis.

Reference Unit bacterial isolates were identified to genus/species level by Matrix-Assisted Laser Desorption/Ionization Time-of-Flight (MALDI-ToF) mass spectrometry (Bruker Daltonics, Bremen, Germany). Methodologies to determine MICs were BSAC agar dilution, broth microdilution or gradient strip testing, and results were interpreted according to EUCAST clinical breakpoints [15]. Carbapenemase genes were sought by real-time PCR in all isolates submitted for investigation of carbapenem resistance and/or where the antibiogram suggested carbapenemase production. Variable-number tandem repeat (VNTR) analysis was performed on selected *K. pneumoniae* isolates to support outbreak investigations as previously described [16] and used to infer common sequence types (STs). 

### Antimicrobial usage data source

Information on ceftazidime/avibactam and colistin (a last line antibiotic comparator) usage in English NHS Trusts, hospitals under the same management, was obtained from IQVIA for 1 March 2017–31 March 2020. The IQVIA Hospital Pharmacy Audit (HPA) database contains information on dispensed drugs from all acute NHS Trust pharmacy systems in England. Hospital admission data were taken from Hospital Episode Statistics (HES) published by NHS England [17].

### Data analyses

The bacterial species being tested for ceftazidime/avibactam susceptibility in routine surveillance data were assessed and trends in incidence and resistance were analysed further for Enterobacterales species and *P. aeruginosa*. Resistance was presented as percentages, rate per 1,000 hospital admissions, or as MICs. Exact binomial confidence intervals with 95% confidence (95% CIs) were calculated. Ceftazidime/avibactam usage data were presented as defined daily doses (DDDs; from the World Health Organization’s DDD index [18]), and as DDD’s per 1,000 hospital admissions.

The analysis in the paper describes early ceftazidime/avibactam usage since 1 March (month of launch) 2017, as well as resistance trends since 1 April 2016, before the introduction of the subscription model; as such, data after 1 April 2020 have been excluded from this analysis. This also limits the potential impact of the COVID-19 pandemic on the results.

## Results

### Routine susceptibility testing and resistance descriptive trends

**Figure 1:**
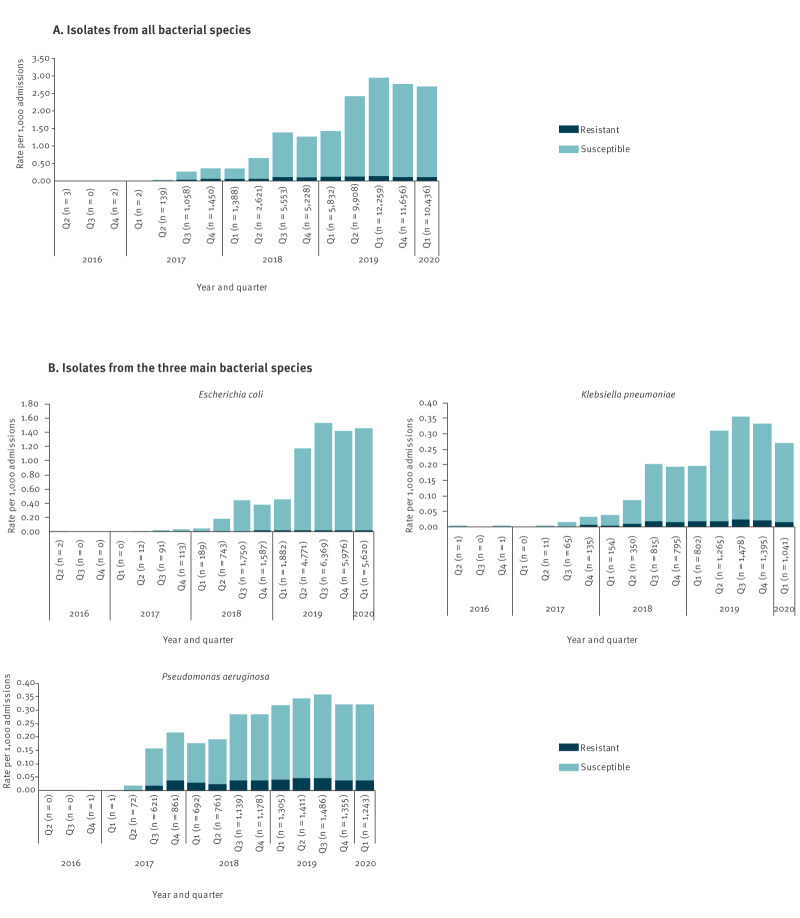
Hospital admission rate of infections with bacteria tested for susceptibility to ceftazidime/avibactam by (A) all and (B) main bacterial species, based on routine diagnostic-laboratory surveillance data, England, April 2016–March 2020 (n = 67,535^a^ bacterial isolates)

Source: SGSS routine diagnostic laboratory surveillance data.

Laboratory records of SGSS including routine susceptibility test results for ceftazidime/avibactam increased from five records reported by three diagnostic laboratories in 2016 (following the antibiotic’s approval by the EMA in April 2016) to 10,436 records reported by 53 diagnostic laboratories in the first quarter of 2020 (Figure 1).

Q1: January to March; Q2: April to June; Q3: July to September; Q4: October to December.

^a^ This excludes 14 specimens from non-bacterial species that were reported with a ceftazidime/avibactam susceptibility test result.

Panel A presents the susceptibility test rates per 1,000 admissions for ceftazidime/avibactam over time for all bacterial isolates, showing apparent gradual increases in the total number isolates tested year on year and low levels of resistance being recorded. Panel B presents the ceftazidime/avibactam trends in testing and resistance rates per 1,000 admissions for *Escherichia coli*, *Klebsiella pneumoniae* and *Pseudomonas aeruginosa*, the top three reported species with ceftazidime/avibactam susceptibility testing results recorded.

Between April 2016 and March 2020, 67,549 specimens from 51,949 patients were tested for ceftazidime/avibactam susceptibility in England, most patients having one specimen (82.4%; 42,820) but nonetheless 67 patients with over 20 (0.1%) reported in the time period. The median age of these patients was 64 years (inter-quartile range (IQR): 41 to 77 years), 43.1% were male (22,399/51,949), 56.8% were female (29,503/51,949), and sex was unknown for 47 patients.

Overall, 11% of reported specimens were from sterile sites (7,397/67,549) and 20% were screening samples (13,182/67,549). Ninety-four per cent of tested specimens were Enterobacterales or *P. aeruginosa* (63,552/67,549)*,* with *E. coli* (29,105/67,549; 43.1%; 95%CI: 42.7–43.5%), *P. aeruginosa* (12,126/67,549; 18.0%; 95%CI: 17.7–18.2%), *K. pneumoniae* (8,308/67,549; 12.3%; 95%CI: 12.1–12.5%) and *Enterobacter cloacae* complex (4,451/67,549; 6.6%; 95%CI: 6.4–6.8%) accounting for 80% (53,990/67,549) as described in Supplementary Table S1. Fourteen tested specimens were reported as being from non-bacterial species (0.02%).

Resistance rates differed by species and over time (Figure 1). Overall, 6.3% of Gram-negative bacteria tested for ceftazidime/avibactam susceptibility were found to be resistant (4,200/66,914; 95%CI: 6.1–6.4%). For Enterobacterales, 3.5% (1,825/51,426; 95%CI: 3.4–3.7%) of isolates tested were reported to SGSS as phenotypically resistant to ceftazidime/avibactam. For *E. coli*, this was 1.8% (532/29,105; 95%CI: 1.7–2.0%), for *P. aeruginosa* 13.3% (1,617/12,126; 95%CI: 12.7–14.0%), for *K. pneumoniae* 7.5% (626/8,308; 95%CI: 7.0–8.1%) and for *E. cloacae* complex 9.0% (400/4,451; 95%CI: 8.2–9.9%). Changes over time in percentage resistance were noted, with higher percentages observed in the early years, primarily because of selective testing and very few laboratories looking at susceptibility to ceftazidime/avibactam, moving to more stable percentage resistance in each pathogen in 2019 and 2020, when the testing of isolates for ceftazidime/avibactam susceptibility increased in number. Comparing the second and final year resistance rates, for *E. coli*, 8.1% were resistant between April 2017 and March 2018 (33/405; 95%CI: 5.7–11.3%) reducing to 1.3% *E. coli* (288/22,736; 95%CI: 1.1–1.4%) between April 2019 and March 2020; similarly, for *P. aeruginosa*, 15.3% (344/2,246; 95%CI: 13.9–16.9%) to 12.6% (690/5,495; 95%CI: 11.7-13.5%), and for *K. pneumoniae*, from 14.5% (53/365; 95%CI: 11.1–18.6%) to 6.1% (314/5,179; 95%CI: 5.4–6.7%). Concurrent carbapenemase mechanism testing data were not available within the SGSS laboratory reports to identify metallo-carbapenemase-positive isolates, which are expected to be ceftazidime/avibactam resistant.

### AMRHAI Reference Unit susceptibility testing and molecular findings

**Figure 2:**
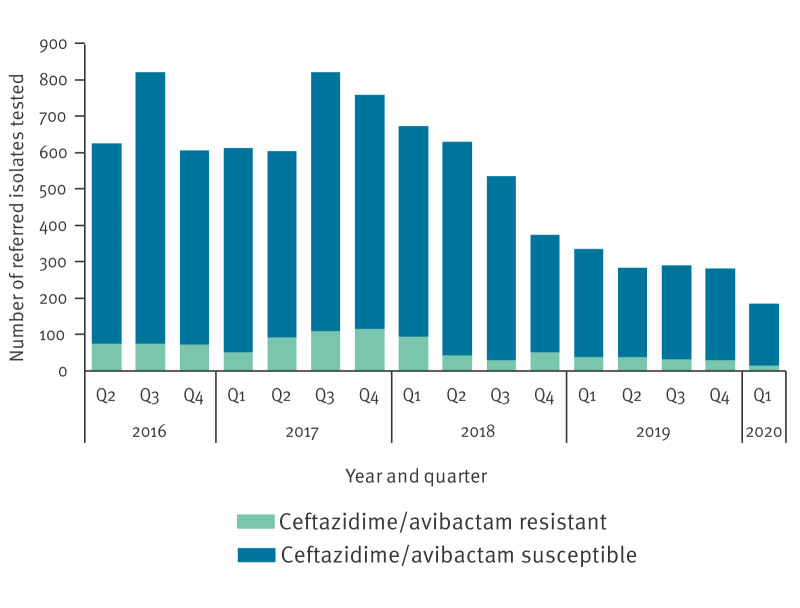
Number of Enterobacterales for which ceftazidime/avibactam MIC was determined including the number of resistant, UKHSA AMRHAI Reference Unit, England, April 2016–March 2020 (n = 8,437 isolates tested)

Source: AMRHAI Reference microbiology Unit.

Between 1 April 2016 and 31 March 2020, the AMRHAI Reference Unit determined ceftazidime/avibactam MICs for 8,437 Enterobacterales, 794 were from sterile sites (blood/cerebrospinal fluid; 9.4%; 95%CI: 8.8–10.1%) and 2,233 were screening samples (26.5%; 95%CI: 25.5–27.4%). The most frequently identified species was *K. pneumoniae* (33.4%; 2,820/8,437; 95%CI: 32.4–34.4%), followed by *E. coli* (29.1%; 2,454/8,437; 95%CI: 28.1–30.1%); overall 1,793 (21.3%; 95%CI: 20.4–22.1%) Enterobacterales were confirmed as harbouring one or more carbapenemase genes (Figure 2 and Table 1).

AMRHAI: antimicrobial resistance and healthcare associated infections; MIC: minimum inhibitory concentration; Q1: January to March; Q2: April to June; Q3: July to September; Q4: October to December; UK: United Kingdom; UKHSA: UK Health Security Agency.

In 2019 the referral criteria changed; these changes have led to a reduction in overall referred isolates and a move to an increased percentage of sterile-site samples.

**Table 1:** Ceftazidime/avibactam MICs for carbapenemase-producing Enterobacterales, UKHSA AMRHAI Reference Unit, England, April 2016–March 2020 (n = 1,793 isolates tested)

Carbapenemase^a^ (n = 1,793)	Ceftazidime/avibactam MIC (mg/L)									Susceptible^b^ isolates		Total number of isolates
	≤ 0.25	0.5	1	2	4	8	16	32	> 32	Number	%	
**Ambler class A (non-metallo-β-lactamases)**												
KPC (251)	80	79	61	20	7	2	0	1	1	249	99.2	251
GES (10)	1	2	1	4	2	0	0	0	0	10	100.0	10
IMI (13)	4	4	4	1	0	0	0	0	0	13	100.0	13
SME (2)	1	1	0	0	0	0	0	0	0	2	100.0	2
FRI-2 (1)	1	0	0	0	0	0	0	0	0	1	100.0	1
**Class D (non-metallo-β-lactamases)**												
OXA-48-like (612)	312	141	110	34	7	2	0	3	3	606	99.0	612
**Class B (metallo-β-lactamases)**												
NDM (598)	4	0	0	3	1	1	2	2	585	9	1.5	598
VIM (25)	0	1	0	0	2	3	3	5	11	6	24.0	25
IMP (157)	1	0	0	0	0	2	2	4	148	3	1.9	157
**Multiple, non-metallo-β-lactamases only**												
KPC + OXA-48-like (19)	0	0	1	0	1	6	11	0	0	8	42.1	19
**Multiple, including metallo-β-lactamase**												
OXA-48-like + NDM (95)	1	0	1	0	0	0	0	0	93	2	2.1	95
OXA-48-like + VIM (1)	1	0	0	0	0	0	0	0	0	1	100.0	1
OXA-48-like + IMP (2)	0	0	0	0	0	0	0	0	2	0	0.0	2
KPC + VIM (3)	0	0	0	0	0	0	1	0	2	0	0.0	3
KPC + OXA-48-like + IMP (1)	0	0	0	0	0	0	0	0	1	0	0.0	1
KPC + NDM (1)	0	0	0	0	0	0	0	0	1	0	0.0	1
IMP + NDM (1)	0	0	0	0	0	0	0	0	1	0	0.0	1
VIM + NDM (1)	0	0	0	0	0	0	0	0	1	0	0.0	1
**Number of isolates**	**406**	**228**	**178**	**62**	**20**	**16**	**19**	**15**	**849**	**910**	**50.8**	**1,793**

AMRHAI: Antimicrobial Resistance and Healthcare Associated Infections; EUCAST: European Committee on Antimicrobial Susceptibility Testing; MIC: minimum inhibitory concentration; UK: United Kingdom; UKHSA: UK Health Security Agency*.*

^a^ FRI: French imipenemase; GES: Guiana extended-spectrum; IMP: imipenemase; KPC: *Klebsiella pneumoniae* carbapenemase; NDM: New Delhi metallo-β-lactamase; OXA: oxacillinase; SME: *Serratia marcescens* enzyme; VIM: Verona integron-mediated metallo-β-lactamase.

^b^ MIC cut off for resistance is > 8mg/L based on EUCAST v10.0 (2020) breakpoints [15].

Overall, among the 8,437 isolates, 968 (11.5%; 95%CI: 10.8–12.2%) were resistant to ceftazidime/avibactam. Of these 968, 864 (89.3%; 95%CI: 87.1–91.1%) were accounted for by the presence of MBL genes, 19 (2.0%; 95%CI: 1.2–3.0%) for that of non-metallo-carbapenemase genes (but negative MBL) while 85 (8.8%; 95%CI: 7.1–10.7%) tested carbapenemase-negative.

Of note, irrespective of resistance status, the 8,437 isolates comprised 1,793 which were positive for one or more carbapenemase genes, 885 included an MBL gene (49.4%; 95%CI: 47.0–51.7%). Of these, 97.6% (864/885; 95%CI: 96.4–98.5%) presented with ceftazidime/avibactam resistance. In contrast, only 2.1% (19/908; 95%CI: 1.3–3.2%) of isolates positive for one or more non-metallo-carbapenemase genes (but negative for metallo-carbapenemase genes) were resistant (Table 1). Among these, 11/19 (57.9%; 95%CI: 33.5–79.7%) isolates were part of an outbreak associated with *K. pneumoniae* sequence type (ST) 258 harbouring *bla*_KPC_ and *bla*_OXA-48-like_ genes [19], six isolates (three *K. pneumoniae*, two *E. coli* and one *Citrobacter freundii*) harboured a *bla*_OXA-48-like_ gene and two *K. pneumoniae* harboured a *bla*_KPC_ gene. The remaining 85 ceftazidime/avibactam-resistant carbapenemase-negative isolates had antibiograms that were suggestive of ESBL and/or AmpC with or without porin loss (n = 53; 62.4%; 95%CI: 51.2–72.6%) or varied MIC profiles characterised by a lack of cephalosporin/clavulanate or cefotaxime/cloxacillin synergy (n = 32; 37.6%; 95%CI: 27.4–48.8%).

The underlying ceftazidime/avibactam resistance mechanism was investigated in one instance for one patient in 2018. For this patient, emergent ceftazidime/avibactam resistance was suspected when, 6 weeks after a ceftazidime/avibactam susceptible result, a resistant isolate was identified while on treatment. Three *bla*_KPC_ isolates were investigated further. All three isolates belonged to a VNTR profile that was consistent with ST258; however, sequencing of the *bla*_KPC_ amplicon identified *bla*_KPC-33_ in the ceftazidime/avibactam-resistant isolate and *bla*_KPC-2_ in a representative ceftazidime/avibactam susceptible isolate.

### Ceftazidime/avibactam usage rates of in hospital settings

**Figure 3:**
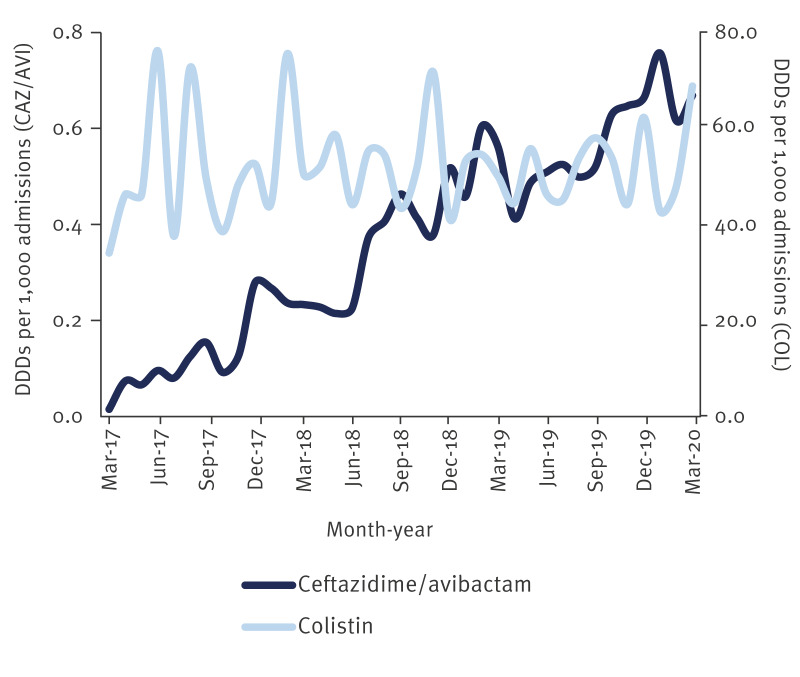
Ceftazidime/avibactam and colistin use in DDDs per 1,000 admissions in NHS Trusts by month, England, March 2017–March 2020

Ceftazidime/avibactam was used by 69.5% (105/151) of English NHS Trusts since the antibiotic’s launch in the UK in March 2017 and national monthly usage increased from 21 DDDs in March 2017 (0.01 DDD/1,000 admissions) to 744 DDDs in March 2020 (0.67 DDD/1,000 admissions) (Figure 3). 

CAZ/AVI: ceftazidime/avibactam; COL: colistin; DDD: defined daily doses; NHS: National Health Service.

Six specialties combined (respiratory medicine, intensive/critical care, general surgery, paediatrics, general medicine and haematology) used approximately two-thirds of the total DDDs (12,421/18,391 DDDs), with some change over time (926/1,448 in 2017, 3,214/5,303 in 2018, 6,491/8,995 in 2019). While lower in overall DDDs (2,457/18,391 DDDs), intensive/critical care patients received the most ceftazidime/avibactam per admission (54.2 DDD/1,000 admission), as outlined in the Table 2.

**Table 2:** Ceftazidime/avibactam usage in NHS Trusts by specialty expressed in DDDs per 1,000 hospital admissions by quarter, England, Quarter 1 2017–Quarter 1 2020

Specialty	Defined daily doses (DDDs) per 1,000 admissions													
	2017				2018				2019				2020	Total
	Q1	Q2	Q3	Q4	Q1	Q2	Q3	Q4	Q1	Q2	Q3	Q4	Q1	
Respiratory Medicine	0.16	0.40	1.06	0.40	0.95	1.18	2.39	4.47	7.41	6.68	7.25	9.50	7.28	3.92
Intensive/critical care medicine	3.27	23.31	35.11	18.78	45.53	74.03	98.58	55.13	94.50	50.18	57.94	58.20	66.36	54.18
General surgery	0.00	0.10	0.26	0.23	0.04	0.05	0.20	0.39	0.65	0.38	0.22	0.64	0.83	0.31
Paediatrics	0.00	0.04	0.03	0.19	0.34	0.14	0.33	0.25	0.49	0.68	1.02	0.66	0.67	0.37
General medicine	0.00	0.00	0.01	0.13	0.14	0.17	0.25	0.19	0.14	0.12	0.16	0.33	0.43	0.16
Haematology	0.00	1.88	1.39	4.57	8.42	4.63	9.47	4.78	4.34	11.07	9.90	7.38	8.20	5.95
Other	0.00	0.05	0.05	0.10	0.16	0.12	0.25	0.28	0.22	0.20	0.23	0.30	0.34	0.18
**TOTAL**	**0.01**	**0.08**	**0.12**	**0.16**	**0.24**	**0.22**	**0.41**	**0.43**	**0.54**	**0.47**	**0.51**	**0.64**	**0.68**	**0.35**

DDD: defined daily doses; NHS: National Health Service; Q1: January to March, Q2: April to June, Q3: July to September, Q4: October to December*.*

## Discussion

In England, a subscription model was launched in December 2020 to incentivise novel antibiotic development and to preserve the selected antibiotics for use in the years to come [20]. For future assessment of this model, and to provide a baseline to further monitor levels of resistance to these antibiotics and their usage, the current work assessed available data for ceftazidime/avibactam, one of the selected antibiotics, before the model launch. By restricting our study to resistance and use before the end of March 2020, our results also avoid including data from the COVID-19 pandemic when healthcare delivery and testing capacity were severely impacted and therefore not representative [21]. Cefiderocol, another novel antibiotic selected for the model, was not included within our analysis, since it was not approved for use in the UK until April 2020, during the first wave of the COVID-19 pandemic [22].

Our investigation of routine diagnostic and reference microbiology laboratory testing data highlights the presence of ceftazidime/avibactam resistance in Enterobacterales in England, albeit at modest levels (<12% in samples tested). However, almost 90% of resistance confirmed at the national reference laboratory was associated with strains producing MBL, which are known to confer ceftazidime/avibactam resistance. 

Prior to April 2019, the UKHSA’s routine AMR surveillance did not capture carbapenemase identification test results performed at diagnostic laboratories. However, from April 2019 onwards routine surveillance reporting was updated to enable capture of results from local diagnostic laboratory carbapenemase identification tests and to inform interpretation of reported carbapenem and therefore also ceftazidime/avibactam resistance. In addition, the Health Protection regulations were updated in October 2020 to include acquired carbapenemase-producing Gram-negative bacteria isolated from human specimens as notifiable, since which time laboratories in England have been required to report cases to the UKHSA [21,23]. A survey of laboratories in the year after becoming notifiable indicated that 88% of laboratories in England could detect the ‘big-4’ carbapenemase(s) (i.e. KPC, NDM, OXA-48-like and VIM) [24], and 88% NHS Trusts reported that they undertook carbapenemase screening [25], suggesting that future monitoring of emerging ceftazidime/avibactam resistance using routine data would be possible. In contrast, isolate referral to the national reference laboratory reflects specimens from more complex patients, including cases where treatment failure may have occurred.

One study limitation is the lack of prescribing information at individual patient level. However, from one patient ceftazidime/avibactam-resistant and susceptible *bla*_KPC_-producing *K. pneumoniae* ST258 isolates were referred (data not shown), with the ceftazidime/avibactam-resistant isolate harbouring *bla*_KPC-33_, which has previously been associated with ceftazidime/avibactam resistance and increased susceptibility to carbapenems [26]. Follow-up revealed that the patient had been repatriated following hospitalisation abroad where ceftazidime/avibactam had been administered. Upon return to the UK, the patient underwent further ceftazidime/avibactam treatment during which the ceftazidime/avibactam-resistant organism was isolated. Treatment with ceftazidime/avibactam has been shown previously to lead to development of resistant subpopulations of *K. pneumoniae* [27,28]. As of January 2020, the real-time PCR used within the AMRHAI Reference Unit to screen putative carbapenemase-producers not only detects *bla*_KPC_ (and other families of carbapenemase genes) but is also able to detect *bla*_KPC_ genes harbouring the point mutation leading to the Asp179Tyr mutation commonly but not exclusively associated with ceftazidime/avibactam resistance [29,30], thus allowing for routine surveillance. Isolates that lack one of these *bla*_KPC_ mutants but exhibit ceftazidime/avibactam resistance are also further characterised by whole-genome sequencing to understand the underlying resistance mechanism(s) and their propensity for further dissemination.

Going forward, laboratory reports should be linked to patient-level medical records including prescribing, adherence to treatment guidelines [6], and clinical data to allow analysis of the indications for prescribing and outcome of treatment. Internationally, documentation of confirmed colonisation or infection by ceftazidime/avibactam-resistant Enterobacterales in case of cross-border patient transfer, as well as timely sharing of outbreaks via electronic early-warning platforms would strengthen prevention of international spread of these organisms [3].

To our knowledge, our study is the first national review assessing ceftazidime/avibactam resistance and carbapenemase profile in routine diagnostic specimens in England. Other studies have modelled the broader impact of using ceftazidime/avibactam in clinical practice [31], acknowledging that restricting use limits the development of resistance, however, at the expense of increasing resistance to other Gram-negative therapeutic options.

Antibiotic usage data from NHS Trusts show that during the study period ceftazidime/avibactam use in the clinical setting was still low in England, but steadily increased since its launch in the UK in 2017. In 2018, more than half of the DDDs were used for treatment of patients on intensive/critical care, or who were in respiratory/general medicine, haematology, paediatric and general surgery wards, although, per patient admission, intensive/critical care accounted for the highest usage rates. Historically, colistin has been one of a few last-resort antibiotics for the treatment of CRE. Due to the agent’s toxicity and suboptimal pharmacokinetics, it is encouraging to see that recent studies comparing colistin with newly approved antibiotics show better clinical outcomes for agents such as ceftazidime/avibactam [32-34]. As our findings show, ceftazidime/avibactam is increasingly being used in England and should be considered as a first-line treatment for CRE infections due to Gram-negative bacteria producing non-metallo-carbapenemase(s).

Our study also highlights the importance of carbapenemase gene detection and variant identification before ceftazidime/avibactam use to inform appropriate treatment options and improve patient outcomes [35]. While not assessed in this study, combination therapy and co-resistance may also prove to be important to investigate [36]. European guidelines do not currently recommend combination therapy for treating CRE susceptible to ceftazidime/avibactam [37], and despite favourable microbiological outcomes clinical benefits were not significantly improved with combination therapy [38], however future evaluations may consider this a potential option.

Limitations of our study include, that while it benefits from being derived from a national surveillance system with good coverage across England, analysis is done at whole-population and country level. Yet, regional and demographic based differences in carbapenemase distribution and carriage across England are known, with some areas having high rates of MBL(s) [39]. Additionally, testing coverage varies between laboratories and clinical settings. Testing of ceftazidime/avibactam in a diagnostic laboratory will more likely reflect intention to use this therapeutic agent rather than the standard local antibiotic testing panel, although some laboratories will test more extensively. The testing and usage reflect similar increasing trends over time; however, the surveillance and prescribing data do not include clinical information or therapeutic indication for the ceftazidime/avibactam use.

Moreover, our dataset is biased towards isolates from more complicated infections, isolate referral to the AMRHAI Reference Unit is voluntary and the isolate referral guidance changed during 2019, leading to a drop in referral for carbapenemase identification and MIC determination [40] which could be further explored through sensitivity analysis. However, by assessing alongside the diagnostic laboratory routine surveillance data for changes, and with the increase in local carbapenemase gene screening and testing [24,25,39], monitoring of emerging ceftazidime/avibactam resistance will mitigate this prospectively.

## Conclusions

During our study period, an instance of emergence of ceftazidime/avibactam resistance during treatment was identified in England, amid increases in microbial diagnostic testing and usage. Individualised treatment based on microbiology testing results and clinical risk factors is recommended. The findings also emphasise the importance of vigilance by clinicians and microbiologists working in clinical specialties where ceftazidime/avibactam is used to detect emerging resistance and prevent further spread within healthcare settings. Detection of CRE that are ceftazidime/avibactam resistant and do not produce an MBL should be referred to a reference laboratory for further investigation [14]. Further robust research is needed now that carbapenemase testing has been made notifiable to determine whether the subscription model is having an impact on usage and resistance.

## Supplementary Data

245000 SupplementaryMaterial
