# Supplementary material for: The importance of monitoring a new antibiotic: ceftazidime/avibactam usage and resistance experience from England, 2016 to 2020
Source: Euro Surveill. 2025 Apr 10;30(14):2400399. doi: 10.2807/1560-7917.ES.2025.30.14.2400399 (PMC11987491; doi:10.2807/1560-7917.ES.2025.30.14.2400399)
Supplement: SupplementaryMaterial [file 2400399_SupplementaryMaterial_TableS1.pdf]

Supplementary material

This supplementary material is hosted by *Eurosurveillance* as supporting information alongside the article *"The importance of monitoring a new antibiotic: Ceftazidime/avibactam usage and resistance experience from England, 2016 to 2020"*, on behalf of the authors, who remain responsible for the accuracy and appropriateness of the content. The same standards for ethics, copyright, attributions and permissions as for the article apply. Supplements are not edited by *Eurosurveillance* and the journal is not responsible for the maintenance of any links or email addresses provided therein.

Data presented here provides additional detail on the species identified with ceftazidime/avibactam resistance from the routine diagnostic laboratory data in England. Data sources are described fully within the accompanying manuscript.

Table S 1. Ceftazidime/avibactam susceptibility test results by species from routine laboratory specimens, England, January 2016 to March 2020

**Species and species group**	**Distribution of ceftazidime/avibactam tests**				**Ceftazidime/avibactam resistance**			
	**Number tested**	**Percentage of total (%)**	**Lower 95% CI (%)**	**Upper 95% CI (%)**	**Number**	**Percentage (%)**	**Lower 95% CI (%)**	**Upper 95% CI (%)**
Escherichia coli	29105	43.1	42.7	43.5	532	1.8	1.7	2.0
Pseudomonas aeruginosa	12126	18.0	17.7	18.2	1617	13.3	12.7	14.0
Klebsiella pneumoniae	8308	12.3	12.1	12.5	626	7.5	7.0	8.1
Enterobacter cloacae complex	4451	6.6	6.4	6.8	400	9.0	8.2	9.9
Citrobacter spp.	2032	3.0	2.9	3.1	112	5.5	4.6	6.6
Proteus mirabilis	1831	2.7	2.6	2.8	10	0.5	0.3	1.0
Klebsiella oxytoca	1086	1.6	1.5	1.7	28	2.6	1.7	3.7
Klebsiella aerogenes	1075	1.6	1.5	1.7	39	3.6	2.6	4.9
Serratia marcescens/ureilytica	926	1.4	1.3	1.5	11	1.2	0.6	2.1
Morganella morganii	899	1.3	1.2	1.4	7	0.8	0.3	1.6
Other Enterobacterales species(a)	1713	2.5	2.4	2.7	60	3.5	2.7	4.5
Other Gram-negative bacterial species	3362	5.0	4.8	5.1	758	22.5	21.3	24.0
Other species(b)	66914	99.1	99.0	99.1	4200	6.3	6.1	6.4
**Total**	**67549**	**NA**	**NA**	**NA**	**4332**	**6.4**	**6.2**	**6.6**

CI= confidence interval.

(a)Species and genera outside of the top ten have been grouped;

(b)includes Gram-positive bacteria, fungi, viruses, amoeba, unknown species (molecular diagnostic test).

Source: English national *routine laboratory surveillance data*
